# Prediction of multi-drug resistance transporters using a novel sequence analysis method

**DOI:** 10.12688/f1000research.6200.2

**Published:** 2015-05-29

**Authors:** Jason E. McDermott, Paul Bruillard, Christopher C. Overall, Luke Gosink, Stephen R. Lindemann

**Affiliations:** 1 Biological Sciences, Pacific Northwest National Laboratory, Washington, WA, 99352, USA; 2 National Security Divisions, Pacific Northwest National Laboratory, Washington, WA, 99352, USA; 3 Department of Molecular Microbiology and Immunology, Oregon Health & Science University, Portland, OR, 97239, USA

**Keywords:** antibiotic resistance, bacteria, machine learning, linguistics, protein function, multidrug resistance transporters, microbiome

## Abstract

There are many examples of groups of proteins that have similar function, but the determinants of functional specificity may be hidden by lack of sequence similarity, or by large groups of similar sequences with different functions. Transporters are one such protein group in that the general function, transport, can be easily inferred from the sequence, but the substrate specificity can be impossible to predict from sequence with current methods. In this paper we describe a linguistic-based approach to identify functional patterns from groups of unaligned protein sequences and its application to predict multi-drug resistance transporters (MDRs) from bacteria. We first show that our method can recreate known patterns from PROSITE for several motifs from unaligned sequences. We then show that the method, MDRpred, can predict MDRs with greater accuracy and positive predictive value than a collection of currently available family-based models from the Pfam database. Finally, we apply MDRpred to a large collection of protein sequences from an environmental microbiome study to make novel predictions about drug resistance in a potential environmental reservoir.

## Introduction

Gram-negative bacteria are a major cause of many human diseases and, due to the emergence of antibiotic resistance, new means to combat them are a pressing international health issue. Recently the Center for Disease Control and Prevention (CDC) highlighted this problem, by stating that, “… new antibiotics will always be needed to keep up with resistant bacteria…” ( CDC, 2013). Antibiotic resistance is mediated by several distinct mechanisms including enzymatic conversion of antibiotics and transporters that eliminate antibiotics from inside cells ( Blair *et al.*, 2015). Transporter superfamilies can be easily identified by standard sequence similarity but specific functional information (e.g. substrate specificity) can be more problematic.

Protein function has traditionally been determined by costly and time-consuming experimental approaches. Tools to determine sequence similarity such as BLAST have enabled efficient annotation of novel proteins by transfer of function. Such methods have been very effective at delineating families of functionally similar proteins that have similar sequences. More flexible approaches using simple grammars like regular expressions and hidden Markov models have improved this process significantly ( Bateman *et al.*, 2000; Gough & Chothia, 2002). However, there remain many proteins that cannot be readily associated with known functions using these approaches, largely because they are unrelated by sequence. The field of linguistics is concerned with the structure of languages and studies morphology, syntax, and semantics. This task, which is grounded in mathematics, is directly analogous to the task of interpreting sequences of amino acids to predict function. To date, the application of linguistic-rooted approaches, such as generative grammars, to protein sequences and the use of rigorous and exhaustive approaches to optimize models has been limited.

Generative grammars have a rich history in linguistic analysis with limited application to biological problems ( Durbin *et al.*, 1998). They can be classified in terms of the Chomsky hierarchy where grammars lower in the hierarchy (e.g., regular grammars) are simpler to understand, compute with, and parse; while grammars further up in the hierarchy are more complex but also have more descriptive power. Algorithms such as PROSITE ( Hofmann *et al.*, 1999) identify simple motifs in proteins using regular expressions, which are the simplest form of grammar (i.e. regular grammars). Hidden Markov models (HMM), a type of regular grammar, have also been applied to detect protein motifs and families. In addition to regular grammars, computational biologists have utilized stochastic context-free grammars for sequence modeling ( Anderson *et al.*, 2012; Dyrka *et al.*, 2013). Such grammars are better at modeling palindromic sequences that are found in RNA structure. All three of these are limited, however, because they still require an underlying sequence alignment.

The regular expressions contained in the PROSITE database are identified using a manual process to first gather a set of examples of a functional class, perform a multiple sequence alignment on those examples, and finally generate a regular expression by looking at regions of the sequence that align and are generally functionally important, for example a phosphorylated residue or active site. A similar procedure is used to create hidden Markov models (HMMs) such as those found in the Pfam database, except that the process of determining a model is automated. Motif determination using these methods is practically limited to operation on families of related protein sequences that have been aligned and has been carried out manually for individual protein motifs (such as in the PROSITE database). Many proteins with the same function may not have significant sequence similarity to allow alignments to be easily or accurately performed. The dependence on multiple sequence alignments and manual construction of protein patterns limits the ability to provide insight into problematic protein motifs.

Previously we have described an effective approach to classification of problematic protein families such as bacterial type III secreted effectors that share little sequence similarity ( McDermott *et al.*, 2011; Samudrala *et al.*, 2009). This method used a support vector machine to integrate different sequence-based features and did not use multiple sequence alignment; rather, because the secretion signal is located in the most N-terminal region of the proteins, it took advantage of this natural alignment of disparate sequences. For problematic protein families in which the discriminating motifs are located in different regions of the protein, methods are needed to be able to automatically identify motifs or features, even where the sequence background might be very noisy and traditional methods for aligning sequences based on evolutionary conservation will not be effective.

In this study we describe an application of the Proactive Intelligent Learning with Grammar (PILGram) method to protein sequences to develop patterns that can discriminate functional classes of proteins in an alignment-free manner. PILGram uses a genetic algorithm to automate feature selection and build regular expressions that discriminate between classes. We first show that PILGram is able to partially re-create PROSITE patterns for ser/thr phosphatase binding and for zinc fingers in an automated and alignment-free manner. We then apply PILGram to classify transporters involved in drug resistance from other transporter proteins and show that the resulting PILGram model performs better than existing HMM models at classifying proteins in this important functional class. Finally, we combine different PILGram models using a simple voting method to develop an effective classifier called MDRpred. The patterns identified by PILGram map to regions that are likely to be important for substrate specificity, highlighting regions that could be targeted for drug development. We show that PILGram can be a general tool for development of simple patterns for functional classification of protein sequences. As a demonstration we apply MDRpred to a metagenome from an environmental microbial community and highlight several high-confidence predictions of novel MDR transporter proteins. Our results indicate that PILGram may be very effective at identifying functional sequence patterns from groups of protein sequences in the absence of any kind of sequence alignment.

## Methods

### Protein pattern datasets for proof-of-concept

To examine the ability of PILGram to identify patterns from unaligned protein sequences we used sets of sequences used to define regular expressions for protein motifs from the PROSITE database. In this way we could compare the output of PILGram with the established PROSITE patterns that had been generated from the aligned set of protein examples. Proteins matching each indicated PROSITE pattern (positive examples) were obtained from the PROSITE website ( http://prosite.expasy.org) as the “prosite.dat” file. UniProt identifiers were extracted from the “DR” fields and the matching sequences, obtained from the UniProt database, were listed as true positives “T”. Of the sequences in the UniProt database that did not match the positive examples, approximately 6000 were chosen at random (specific numbers given for each example) to serve as negative examples (See PROSITE_positives_PS000125.fasta, PROSITE_negatives_PS000125.fasta, PROSITE_positives_PS00028.fasta, PROSITE_negatives_PS00028.fasta). The most current PROSITE records available at the time were used (See PROSITE_PS00125.txt and PROSITE_PS00028.txt).

### Drug resistance transporter dataset

To construct a training set for multidrug resistance transporters we obtained the protein sequences of 6097 transporter proteins from the Transporter Classification Database [TCDB; ( Saier *et al.*, 2014)] along with family classifications. This database was searched for “drug resistance” giving 71 drug resistance (DR) transporters (See MDR_TCDB_positives.fasta and MDR_TCDB_negatives.fasta datasets). We then searched the protein sequence descriptions from the UniProt database and found an additional 89 sequences annotated with “[drug] resistance” that were not included in the TCDB annotations. We used the TCDB-annotated DR transporters as our positive examples because most are accompanied by references. The ‘candidate’ list of positive examples annotated by UniProt was held out of the training set so as not to interfere with classification. The remaining 5934 sequences were used as negative examples since they are annotated as transporters but not as DR transporters in either database.

### Hot Lake peptide sequences

Metagenomic DNA was extracted from two unicyanobacterial consortia cultivated from a microbial mat inhabiting Hot Lake, WA ( Lindemann *et al.*, 2013) as previously described ( Cole *et al.*, 2014). Metagenome reconstructions were generated as reported by Nelson *et al.*, (manuscript submitted). Briefly, paired-end reads were generated by the US Department of Energy (DOE) Joint Genome Institute (JGI; http://jgi.doe.gov) under CSP 701, quality trimmed using Trimmomatic ( Bolger *et al.*, 2014), and assembled using IDBA-UD ( Peng *et al.*, 2012) with a minimum contig size of 250 bp. Contigs longer than 2 Kb were binned using read coverage for each scaffold using Bowtie2 ( Langmead & Salzberg, 2012) and samtools ( Li *et al.*, 2009). Gene models for the metagenome reconstructions were generated using Prodigal ( Hyatt *et al.*, 2010) and hand-curated in some instances. Additionally, axenic organisms isolated from the consortia were sequenced of 10 Kb libraries with PacBio and assembled by the JGI, also under CSP 701. The genomes of axenic organisms were shown to be identical to the corresponding genome reconstructions in the metagenome (Nelson *et al.*, submitted), and replaced these reconstructions in the metagenome database, being more complete. For the axenic isolates, gene models were generated by IMG/ER ( Markowitz *et al.*, 2009). The sequences are available through NCBI GenBank under accessions, NZ_JQMU00000000.1 GI:675281874 ( *Porphyrobacter* sp. HL-46), NZ_JMMC00000000.1 GI:653087839 ( *Halomonas* sp. HL-48), NZ_JAFX00000000.1 GI:635638184 ( *Algoriphagus marincola* str. HL-49), NZ_JYNR00000000.1 GI:761631804 ( *Marinobacter excellens* HL-55), and NZ_JMLY00000000.1 GI:654325145 ( *Marinobacter* sp. HL-58). Metagenome sequences not mapped to sequences from axenic cultures have been submitted to GenBank and are awaiting accessions.

### Feature generation

Physiochemical properties (PPs) were calculated using the Python propy module ( Cao *et al.*, 2013). Properties were calculated using the 147 Composition, Transition, Distribution (CTD) descriptors in propy ( Dubchak, 1995). Classes of properties include hydrophobicity, normalized van der Waals volume (VDWV), polarity, charge, secondary structure, solvent accessibility, and polarizability. In each class amino acids are grouped into three groups based on their physiochemical properties, for example hydrophobicity includes hydrophobic residues (C, L, V, I, M, F, W), polar residues (R, K, E, D, Q, N), and neutral residues (G, A, S, T, P, H, Y). Groups for other classes can be found in ( Dubchak, 1995). Composition calculates a length-normalized score based on the number of residues in the group (for example polar residues) in the sequence. Distribution calculates the portion of the sequence that includes a certain percentage (1, 25, 50, 75, or 100) of the matches for that group. Transition calculates the number of times an amino acid from one group (polar, e.g.) is found next to one from another group (hydrophobic, e.g.) in the sequence, normalized by length.

The PP-protein regular expressions (PRE) were represented as a combination of regular expression from the standard PRE with one of the PPs. PILGram treats the PP as an independent element to add to a regular expression. The fitness score for a particular combination is evaluated by calculating the PP score (see above) for the region or regions of the sequence matched by the regular expression. If there is more than one matched region by a regular expression the PP scores from each segment are averaged.

As an example if the PRE is “FG*.TL”, then a sequence such as:

MKGGLA **FGADAYLLIWTL**QQST…

would be matched in the underlined region. An additional PP of “hydrophobicityC1” (that is, composition class for hydrophobic amino acids), would be scored by counting the number of hydrophobic residues (C, L, V, I, M, F, W) in the region (6) and dividing by the length of the matched region (12) to give 0.50. A second sequence:

MIYTSSG **FGLLILLYCMTL**RHCN…

would be matched in the underlined region, but the PP hydrophobicityC1 score would be higher 10/12 = 0.83. The PILGram optimization explores many possible combinations using a genetic algorithm (see below) to find the PP and PRE combination that gives the best accuracy.

The transmembrane region (TMR) grammar is composed of the PRE with the addition of predefined patterns that represent potential transmembrane regions. These were established by including all transmembrane regions defined in the entire TCDB ( Saier *et al.*, 2014) with two flanking amino acids from the N- and C-terminal portions of the region, but leaving the sequence of the transmembrane region itself as variable. This means that a given transmembrane region from TCDB (underlined here):

AAQT **LSVYFLAFALGVVIWGVLA**DKWGR

would result in a ‘seed’ TMR-PRE of “QT*.DK”. These seed PREs can then be chosen by PILGram to incorporate into parse trees (see below) to generate new PREs. So the resulting models look identical to those generated by the PRE grammar alone, but may be biased toward a focus on transmembrane regions.

### Performance evaluation

PILGram models were constructed using half the training data and performance was evaluated with the other half. PILGram optimization was based on accuracy:

$$A\;=\;\frac{TP\;+\;TN}{TP\;+\;FP\;+\;TN\;+\;FN}$$

where TP, TN, FP, and FN are true positive, true negative, false positive, and false negative predictions, respectively. For final evaluation of models we also calculated positive predictive value:

$$PPV\;=\;\frac{TP}{TP\;+\;FP}$$

and area under the receiver operator characteristic (ROC) curve (AUC) ( Salzberg, 1997).

### Pattern clustering

Clustering of patterns for MDRpred was accomplished by assembling a vector of binary values (match or no match) across the 6005 examples (71 positive plus 5934 negative examples) from the training set for each of the 36 final MDR patterns. Euclidean distance was calculated between all pairs of vectors and the hcclust function from R (version 3.0.1) was used for hierarchical clustering using complete agglomeration.

### PILGram

Machine learning methods, like SIEVE ( McDermott *et al.*, 2011; Samudrala *et al.*, 2009), take features as input to build a model. Features are the smallest elements derived from the examples (protein sequences) that can be categories (e.g. amino acid type) or values (e.g. solvent accessibility values). While the selection of salient features is critical for classification, most algorithms require their manual specification. PILGram (Proactive Intelligent Learning with Grammar) is an approach to automate the feature selection process and allows for the selection of irredundant features. PILGram does this by combining a genetic algorithm and a generative grammar, which is a formalized set of rules for combining features into different patterns in the form of parse trees. PILGram generates a large number of such trees and then applies a genetic algorithm, which iteratively recombines these trees to determine an optimal model for classification of the positive and negative examples. In this way PILGram specifies an absorbing Markov chain on the space of features, and given sufficient time, will always converge to a collection of optimal non-redundant features. The mathematical foundations of and explicit algorithm for PILGram are currently pending review, but the algorithm is perhaps best understood by example.

Consider the following toy example: height, weight, and age data are gathered from a population and each person is labeled as obese or not. One might like to automate the determination of obesity using only height, weight, and age. It is known that the body mass index (BMI) is a good indicator of obesity and is given by (weight/(height × height)). In order to determine this quantity, PILGram might make use of the following grammar.

〈expr〉::=(〈expr〉〈op〉〈expr〉)|〈attr〉

〈op〉::=+|-| × |/

〈attr〉::=height | weight | age

In this grammar the ‘|’ symbol is to be read as ‘or’ and ‘::=’ can be read as ‘replace by.’ So the second line tells us that ‘〈op〉’ can be replaced by ‘+’, ‘-‘, ‘×’, or ‘/’. The symbols to the left of ::= are called non-terminal symbols. This grammar can be used to generate features as follows.

1. Write down 〈expr〉.
2. Locate any non-terminal symbol in your expression.
3. Replace the chosen non-terminal according to the grammar.
4. If there is a non-terminal symbol in your expression, then return to step 2.

This process can be viewed as a parse tree. That is, at step 1 one writes 〈expr〉. Then every time a non-terminal symbol is replaced one writes the replacement below the non-terminal symbol and connects each symbol in the replacement with the initial symbol with a line. A vertical line is placed below each non-terminal symbol that is not replaced. The resulting expression is then read from left to right along the ‘leaves’ of the resulting tree. For instance, BMI might be produced from the procedure as follows:

〈expr〉→ (〈expr〉〈op〉〈expr〉) → (〈attr〉〈op〉〈expr〉) → (weight 〈op〉〈expr〉) → (weight/〈expr〉) → (weight/(〈expr〉〈op〉〈expr〉)) → (weight/(〈expr〉×〈expr〉)) → (weight/(〈attr〉×〈expr〉)) → (weight/(〈attr〉×〈attr〉)) → (weight/(height ×〈attr〉)) → (weight/(height × height)).

This is more succinctly expressed by the parse tree:

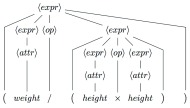



While one might get lucky and generate this expression by random application of the above grammar, it is highly unlikely. However, one might generate (weight-height) and (age+height × height). While neither of these expressions are BMI, BMI can be produced by *mutating* and *crossing* these feature.

Mutation is a process by which a node in the parse tree is randomly selected and then replaced with another value such that the tree remains consistent with the generative rules of the grammar. In some cases, one might opt to re-build the tree below the replaced node thereby giving the algorithm greater flexibility. For instance, the first expression can be represented as a parse tree and mutated as follows:

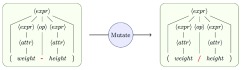



The resulting feature, (weight/height), is more similar to BMI than the initial feature, and in fact performs better at classifying obesity. To arrive at BMI we could apply the *crossing* procedure to (weight/height) and (age+height × height).

Crossing is a process by which two features are expressed as parse trees and two of their subtrees are exchanged so that the resulting parse trees are consistent with the grammar. For instance, BMI can be found by crossing (weight/height) and (age+height × height) as follows:

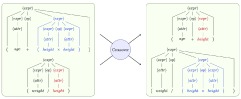



Not all crossings and mutations will produce better features, and not all features should be considered for crossing or mutation. To handle this, PILGram behaves stochastically and preferentially selects features for mutation and crossing according to how well they perform. The guiding principle is that features which perform better should be closer to the optimal feature than those that do not. The entire PILGram algorithm can be outlined as follows:

1. Select a grammar for feature generation and a fitness function to evaluate the features against.
2. Randomly generate a population of features and determine the fitness of each feature.
3. Randomly subsample the population where a feature is selected with probability proportional to its fitness.
4. For each feature selected in step 3, copy the feature and randomly change the value of a random node in its parse tree in a manner consistent with the grammar. Return the initial feature and the result of the mutation to the population.
5. Randomly subsample the population for pairs of features with each feature selected with probability proportional to its fitness.
6. For each pair selected in step 5 produce a copy of their parse trees. Randomly select a subtree in each feature’s parse tree and exchange these subtrees ensuring that the exchange produces features which are consistent with the grammar. Return the two initial features and the two new features to the population.
7. Compute the fitness of all features in the population and remove the least fit features until the population returns to its initial size.
8. If the fittest feature has converged, then terminate the algorithm, otherwise return to step 3.

**Supplemental Figure 1.**
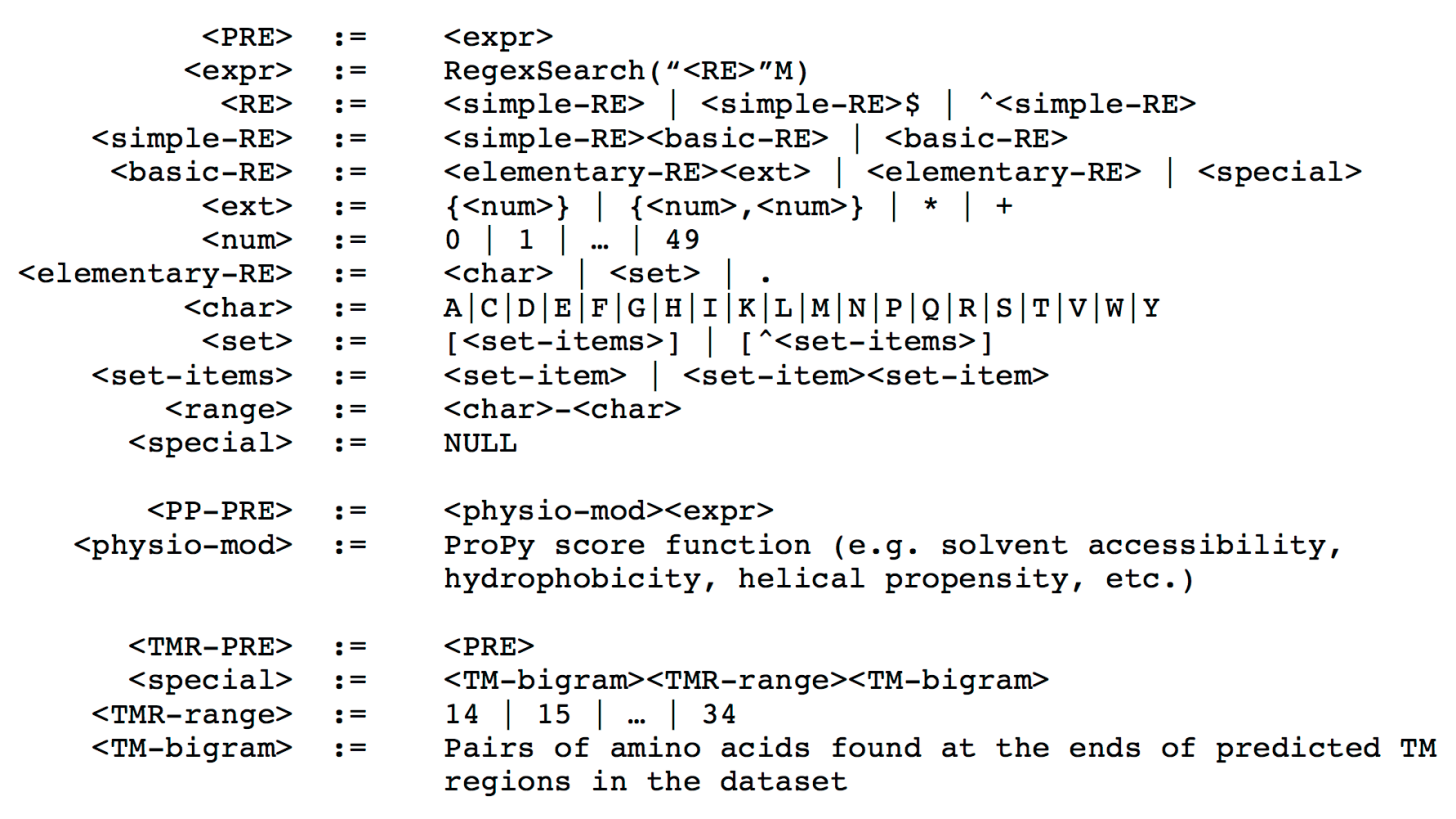
Backus–Naur form grammar for proteins.

A common variation of the algorithm is to randomly generate new features at the start of step 7 and add them to the population before reducing the population size. Another common modification is to iteratively apply the algorithm such that the fitness function is updated between iterations to account for the fittest feature. This allows one to generate a list of irredundant features. Unsurprisingly, the choice of generative grammar strongly influences the quality of the resulting features. Below we will make use of Perl’s regular expression grammar to produce motifs in an alignment free fashion ( Supplemental Figure 1).

Many conventional genetic algorithms use ‘chromosomes’, the group of variables that alter algorithm behavior, with a set length. PILGram is based on the idea of recombining parse trees, so does not have a defined length. As described above the trees can be mutated and crossed during the optimization process, and the length of the resulting regular expression is therefore variable, though it is limited by the maximum depth of parse trees allowed. Parse tree depth can be set but larger values become more computationally intensive.

PILGram has been applied in areas ranging from text analysis, which uses a combination of atomic features based on letter frequency based atomic features and regular expressions, and to image analysis, which uses more complex image-based atomic features. In both of these cases PILGram not only provided features that were optimal for classification, but that were also easily interpreted by a user (unpublished results). In addition to these application spaces, precursor technology has been applied to loop unrolling in the realm of compiler optimization ( Leather *et al.*, 2009) where it was found that learned features resulted in an increase from 48% of the theoretical efficiency bound (using expert driven features) to 76% of the theoretical bound using features automatically identified by a PILGram-like algorithm. We note that PILGram does not train a classifier, rather it selects features which means that any improvements are not the result of overfitting but instead, are a consequence of carefully chosen features.

### Regular expressions

The protein regular expression (PRE) used by PILGram to identify patterns in protein sequences is expressed in standard regular expression notation. Briefly:

**Table:** 

Symbols	Example use	Description
.	.	Matches any single residue
[XYZ]	[AFGHL]	Matches any single residue that is contained in the brackets
[^XYZ]	[^KR]	Matches any single residue that is not contained in the brackets
[X-Y]	[A-E]	Indicates a range of residues in alphabetical order
^	^MST	Matches the start (N-terminus) of the sequence
$	FGH$	Matches the end (C-terminus) of the sequence
X*	A*	Matches zero or more of the preceding element
X+	K+	Matches one or more of the preceding element
X?	C?	Matches zero or one of the preceding element
X{Y}	L{20}	Matches the indicated number of the preceding element
X{Y,Z}	R{2,4}	Matches preceding element Y or Z times

## Results

Prediction of multi-drug resistance transporters dataset Data file 1 Title: Data File PROSITE_positives_PS000125.fasta. Legend: Sequence file in FASTA format of all positive examples for the ser/thr phosphatase model.
Data file 2 Data File PROSITE_negatives_PS000125.fasta. Sequence file in FASTA format of all randomly selected negative examples for the ser/thr phosphatase model."
Data file 3 Data File PROSITE_positives_PS00028.fasta. Sequence file in FASTA format of all positive examples for the zinc finger model.
Data file 4 Data File PROSITE_negatives_PS00028.fasta. Sequence file in FASTA format of all randomly selected negative examples for the zinc finger model.
Data file 5 Data File PROSITE_PS00125.txt. PROSITE record used for the ser/thr phosphatase model.
Data file 6 Data File PROSITE_PS00028.txt. PROSITE record used for the zinc finger model.
Data file 7 Data File MDR_TCDB_positives.fasta. Sequence file of MDR transporters used for training. FASTA format file of positive examples used in this study derived from the TCDB.
Data file 8 Data File MDR_TCDB_negatives.fasta. Sequence file of non-MDR transporters used for training. FASTA format file of negative examples used in this study derived from the TCDB.
Data file 9 Data File PILGram_PATTERNS_PS00125.txt. Regular expression generated by PILGram for the ser/thr phosphatase model.
Data file 10 Data File PS00125_alignments.out. Sequence alignments of PILGram model matches to the positive examples in the ser/thr phosphatase model.
Data file 11 Data File PILGram_PATTERNS_PS00028.txt. Regular expressions generated by PILGram for the zinc finger model.
Data file 12 Data File PS00028_alignments.out. Sequence alignments of PILGram model matches to the positive examples in the zinc finger model and a summary score line that represents the overlap of the 10 different models for each sequence.
Data file 13 Data File PILGram_PATTERNS_MDRpred.txt. The 36 regular expressions and associated physiochemical properties (where applicable) generated by PILGram for the MDR model .
Data file 14 Data File MDRpred_alignments.out. Alignments of 36 PILGram model matches on the MDR positive example sequences.
Data file 15 Data File Pfam_transporters.txt. A list of Pfam families that were used to identify transporters in the Hot Lake metagenome.
Data file 16 Data File HotLake_MDRpred_predictions.fasta. A FASTA format file of 63 protein sequences from the Hot Lake metagenome that are matched by 30 or more MDRpred individual models (high confidence predictions), match Pfam families for transporters (Pfam e-value less than 1e-20), but are not identified by Pfam as multidrug resistance transporters. Click here for additional data file. Copyright: © 2015 McDermott JE et al. 2015 Data associated with the article are available under the terms of the Creative Commons Zero "No rights reserved" data waiver (CC0 1.0 Public domain dedication).

### Alignment-free identification of discriminatory protein patterns in PROSITE

To test the ability of PILGram to identify discriminatory regular expressions from unaligned sequences we focused on a well-defined group of proteins with a known discriminatory pattern. We first examined the serine-threonine phosphatase pattern (PROSITE PS00125) by obtaining 166 sequences listed as true positives from PROSITE (see Methods). For negative examples we randomly selected 5344 sequences from UniProt that are not included in the positive sequences.

We applied PILGram to this dataset using a standard regular expression grammar modified for protein sequences (see Supplemental Figure 1). The algorithm was terminated after 276 iterations when the fitness (classification accuracy) did not change over 10 consecutive iterations. The resulting pattern ( Table 1) had a very high accuracy and positive predictive value (PPV) at 99.9% and 92%, respectively. The pattern identified by PILGram contains the core of the existing PROSITE pattern, a K or R (the PILGram pattern adds a Q) followed by GNH, missing the first and last residue of the PROSITE pattern, and performs nearly as well (See Supplemental Data PILGram_PATTERNS_PS00125.txt). In Table 2 we show several examples of the functional regions identified in sequences by the original PROSITE model and the PILGram-derived model (PRE matches in bold type). Alignments for the complete set of positive examples are included as Supplemental Data PS00125_alignments.out. However, the PILGram pattern required no sequence alignment or manual determination of a conserved pattern.

**Table 1.** Ser/Thr Phosphatase model.

Model	Pattern	Accuracy
*PS00125*	*[LIVMN][KR]GNHE*	*100.0%*
1	[KQR]G+NH	99.9%

The ser/thr phosphatase pattern is relatively simple and does not include any gaps of variable size. We were interested in determining if PILGram would also work on a more complicated pattern and so chose the zinc finger pattern (PS00028), which is a somewhat variable arrangement of conserved cysteine and histidine residues. We obtained the 1997 sequences used for the construction of the PROSITE pattern and additionally collected 5435 randomly selected protein sequences from the UniProt database to serve as negative examples for this test example. Because individual runs converged on different predictive patterns we ran PILGram 10 times on the dataset. In principle, PILGram will always eventually converge to the optimal pattern. However, in practice there may be ‘flat regions’ over which the fitness function does not significantly vary with feature modification or local extrema. In such situations, PILGram may take significant time to escape these regions and it is more economical to employ a weak convergence test, run PILGram several times, and aggregate the features.

**Table 2.** Example alignments of ser/thr phosphatase sequences.

Sequence	Model	Functional region
Q9LHE7	PS00125	PANITL **LRGNHE**SRQLTQ
Q9LHE7	PILGram 1	PANITLL **RGNH**ESRQLTQ
P12982	PS00125	SENFFL **LRGNHE**CASINR
P12982	PILGram 1	SENFFLL **RGNH**ECASINR
A2XN40	PS00125	PQRITI **LRGNHE**SRQITQ
A2XN40	PILGram 1	PQRITIL **RGNH**ESRQITQ

The resulting patterns ( Table 3; Supplemental Data PILGram_PATTERNS_PS00028.txt) vary in composition and accuracy, with a maximum accuracy obtained of about 92%. All patterns fall short of the manually determined PROSITE pattern that has an accuracy of 99%. It is interesting to note that none of the identified patterns perfectly matches any portions of the manually determined PROSITE pattern, though there are some consistently identified features such as multiple cysteine residues.

**Table 3.** Zinc finger patterns identified.

Model	Pattern	Accuracy
*PS00028*	*C.{2,4}C.{3}[LIVMFYWC].{8}H.{3,5}H*	*99.0%*
1	[^LV][^F][^VW]{8}[^ADILN]{7}C	87.6%
2	C[D-H].+[R][^EFG]H	87.4%
3	.{15}C[^FGIRW][^C]C	92.7%
4	[CHP][^V]{53}	81.2%
5	[C].{27}[C]	89.0%
6	[^VW]{55}.*.+$	80.0%
7	C[^V]{42}	83.0%
8	K.{3}C+	87.0%
9	C[AGHNQT]K	87.2%
10	C[^IFPV]{3}F+[^CE]	91.0%
Combined		95.3%

**Figure 1.**
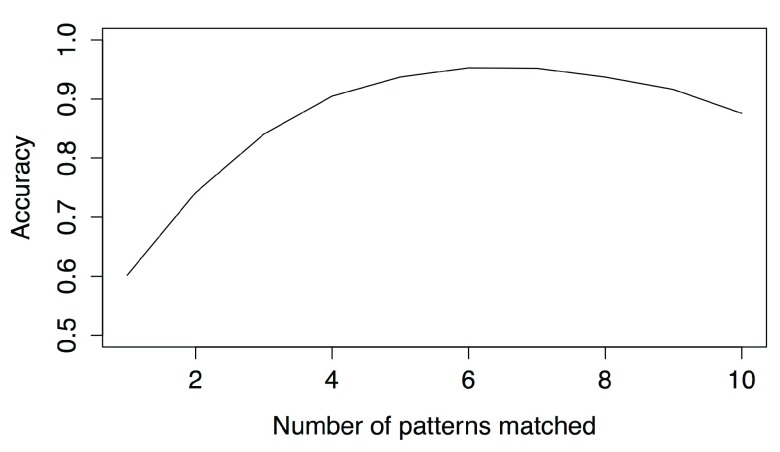
Accuracy for prediction of zinc finger proteins. Matches to PILGram-generated regular expression patterns for the zinc finger domain (represented in PROSITE PS00028) were counted (X axis) and accuracy (Y axis) calculated based on the known positives and negative examples datasets (see text). Peak accuracy of the approach is attained at six pattern matches.

We examined the possibility that the patterns identified by PILGram would be synergistic in their discriminatory ability. For each example protein (positive and negative) we counted how many of the individual PILGram patterns matched, then used this number as a discriminator. We found that using this simple voting procedure increased the accuracy from 92% to a maximum of 95.3% when six or more patterns match a sequence ( Figure 1). While this performance still does not reach the level of the original PROSITE pattern (99%), we believe it demonstrates the utility of PILGram for identifying patterns from unaligned sequences.

We were interested to know if PILGram was identifying regions of the sequence that overlap with the PROSITE pattern. We identified regions in all positive example sequences that match the ten PILGram patterns and calculated a score for each sequence based on the number of matches, per residue, that PILGram identified in the real zinc finger region. On average, 3.4 PILGram patterns match each residue of the known PS00028 pattern, whereas the number of patterns matching arbitrary residues in the sequence was 2.1. This shows that PILGram identifies more patterns overlapping the canonical zinc finger motif. However, it is clear that PILGram-derived motifs may not be canonical and further work needs to be done in this area. We show examples of matches from individual PILGram models as well as the per-residue overlap score (as “Summary”) in Table 4. Note that none of the individual PILGram models matches the single (Q24174, beginning at residue 540) or double (Q59RR0, beginning at residue 645) zinc finger motifs completely, but that the overlap score for the functional regions in both sequences are higher than surrounding sequences. Alignments for the complete set of positive examples are provided as Supplemental Data PS00028_alignments.out.

**Table 4.** Example alignments of zinc finger regions.

Sequence	Model	Functional region
Q24174	PS00028	ATDPRP **CPKCGKIYRSAHTLRTHLEDKH**TVCPGY
Q24174	PILGram 1	ATDPRPCPKCGKIYRSAH **TLRTHLEDKHTVCPGY**
Q24174	PILGram 2	**ATDPRPCPKCGKIYRSAHTLRTHLEDKHTVCPGY**
Q24174	PILGram 3	**ATDPRPCPKC**GKIYRSAHTL **RTHLEDKHTVCPGY**
Q24174	PILGram 4	ATDPRPCPKCGKIYRSAHTLRTHLEDKHTVCPGY
Q24174	PILGram 5	ATDPRPCPKCGKIYRSAHTLRTHLEDKHTVCPGY
Q24174	PILGram 6	**ATDPRPCPKCGKIYRSAHTLRTHLEDKHTVCPGY**
Q24174	PILGram 7	ATDPRPCPKCGKIYRSAHTLRTHLEDKHTVCPGY
Q24174	PILGram 8	ATDPRPCPKCGKIYRSAHTLRTHLED **KHTVC**PGY
Q24174	PILGram 9	ATDPRPCPK **CGK**IYRSAHTLRTHLEDKHTVCPGY
Q24174	PILGram 10	ATDPRPCPKCGKIYRSAHTLRTHLEDKHTVCPGY
Q24174	Summary	222222 3334332222223344444455444333
Q59RR0	PS00028	EDKIYT **CTYKNCGKKFTRRYNVRSHIQTH**LSDRPFG **CQFCPKRFVRQHDLNRHVKGH**IEARYS
Q59RR0	PILGram 1	EDKIYTCTYKNCGKKFTRRYNV **RSHIQTHLSDRPFGCQFC**PKRFVRQHDLNRHVKGHIEARYS
Q59RR0	PILGram 2	**EDKIYTCTYKNCGKKFTRRYNVRSHIQTHLSDRPFGCQFCPKRFVRQHDLNRHVKGHIEARYS**
Q59RR0	PILGram 3	EDKIYTCTYKNCGKKFTRRYN **VRSHIQTHLSDRPFGCQFC**PKRFVRQHDLNRHVKGHIEARYS
Q59RR0	PILGram 4	**EDKIYTCTYKNCGKK**FTRRYNVRSHIQTHLSDRPFGCQFCPKRFVRQHDLNRHVKGHIEARYS
Q59RR0	PILGram 5	EDKIYTCTYKN **CGKKFTRRYNVRSHIQTHLSDRPFGCQFC**PKRFVRQHDLNRHVKGHIEARYS
Q59RR0	PILGram 6	**EDKIYTCTYKNCGKKFTRRYNVRSHIQTHLSDRPFGCQFCPKRFVRQHDLNRHVKGHIEARYS**
Q59RR0	PILGram 7	EDKIYTCTYKNCGKKFTRRYNVRSHIQTHLSDRPFGCQFCPKRFVRQHDLNRHVKGHIEARYS
Q59RR0	PILGram 8	ED **KIYT**CTYKNCGKKFTRRYNVRSHIQTHLSDRPFGCQFCPKRFVRQHDLNRHVKGHIEARYS
Q59RR0	PILGram 9	EDKIYTCTYKN **CGK**KFTRRYNVRSHIQTHLSDRPFGCQFCPKRFVRQHDLNRHVKGHIEARYS
Q59RR0	PILGram 10	**EDKIYTCTYKNCGKKFTRRYNVRSHIQTHLSDRPFGCQFCPKRFVRQHDLNRHVKGHIEARYS**
Q59RR0	Summary	334444544446665444444566666665555555666633333333333333333222222

### Drug resistance transporters

A more difficult task for functional classification is to develop a model that will discriminate a group of functionally related proteins that cannot be aligned by traditional sequence alignment methods, or where the alignment does not allow discrimination between closely related sequences with different functions. To test its utility with these kinds of problematic proteins we applied PILGram to develop a classifier for antibiotic drug resistance transporters.

Though transporter superfamily members can be identified fairly readily using standard sequence alignment approaches, previous studies have shown that sequence similarity has limited utility for classifying of transporters by substrate specificity ( Barghash & Helms, 2013). The same authors also showed separately that integrating simple data (amino acid composition, dipeptide composition) could be used to classify some substrate families with good accuracy ( Schaadt *et al.*, 2010; Schaadt & Helms, 2012), but these models have little potential for providing biological insight. Additionally, it remains unclear if there are members of functional families that have yet to be discovered because of lack of strong sequence similarity. ATP-binding cassette transporters (ABC), resistance-nodulation-cell division (RND) superfamily, and major facilitator superfamily (MFS) transporters are common superfamilies of proteins involved in the transport of a wide variety of different compounds, such as sugars, ions, peptides, and more complex organic molecules. Multidrug resistance (MDR) transporters are found in each of these superfamilies and are primary mediators of antibiotic drug resistance ( Nikaido, 2009; Nikaido & Pages, 2012). Though MDR transporters actually encompass a range of substrate specificities because there are many types of drugs they export, we hypothesized that there would be some unifying features of MDR transporters that could be captured using PILGram.

We gathered a set of 73 known MDR transporter sequences (positive examples) from the TCDB ( Saier *et al.*, 2014) and used the remainder of sequences classified in the TCDB as non-MDR transporters (negative examples; 5935 sequences). This dataset (Supplemental Data MDR_TCDB_positives.fasta and MDR_TCDB_negatives.fasta) was used to train and cross-validate MDRpred as described below.

### Traditional methods of identifying antibiotic resistance transporters

We first evaluated how well previously generated HMM models from the Pfam database could discriminate between MDR and non-MDR transporters. We identified four Pfam models that seem to definitively identify drug resistance transporters (PF00893, PF08370, PF00873, and PF13536) and applied them to the set of sequences considering a ‘hit’ as a sequence matched by any of the models with high confidence (E value < 1e-100). The Pfam models provide very good accuracy (~97%), but only identify 10 of 73 MDR transporters (14%), and these are likely hits to many of the sequences used to create the models in the first place.

### PILGram model training

We examined the ability of PILGram to find patterns capable of identifying MDR transporters from other transporter sequences. Though regular expressions have been shown to be effective at capturing many types of functional patterns in proteins ( Hofmann *et al.*, 1999), other patterns may be more amenable to broader chemical and structural characteristics of regions of proteins ( Dubchak, 1995). Because we believed that transmembrane regions (TMRs) would be important features in this classification task we modified our protein regular expression (PRE) grammar ( Supplemental Figure 1) to bias the feature generation processes toward producing TMRs (TMR-PRE). Additionally, we included a large set of different types of protein physiochemical properties in our PILGram search (PP-PRE). PILGram included the 147 types of properties as features that could be chosen during the search. If a physiochemical property was used in a search the score (value for that particular property) was calculated for all matches of the accompanying regular expression on a sequence. If there were multiple matches to the protein then the scores were averaged.

**Supplemental Figure 2.**
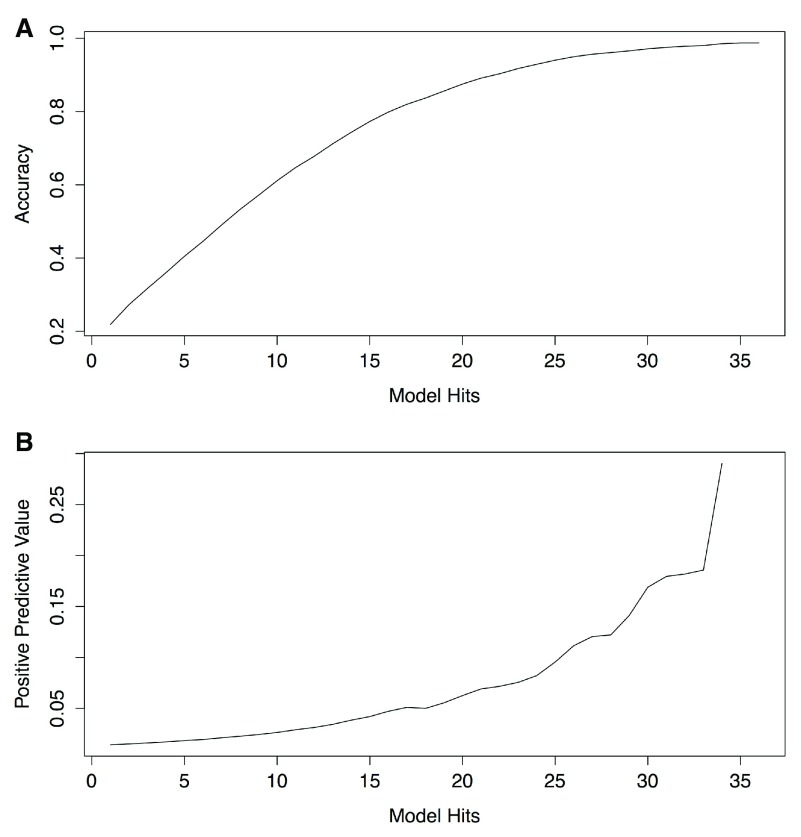
Simple voting method for combining models. **A. Accuracy of combined patterns for classification.** Matches to PILGram-generated regular expression patterns for the MDR transporters were counted (X axis) and accuracy (Y axis) calculated based on the known positives and negative examples datasets (see text). Peak accuracy is attained when all 36 patterns match the sequence, indicating that the diversity of MDR transporter sequences is likely to be high. Redundancy analysis ( Table 3) shows that a similar accuracy can be obtained with seven patterns. **B. Positive-predictive value of combined patterns for classification.** Positive predictive value (the percentage of true positives in all positive predictions; Y axis) was calculated for each number of MDR transporter pattern matches (X axis). The maximum value is reached in sequences that match all patterns.

Using a 2-fold cross-validation approach (see Methods) we used PILGram to generate 36 models ( Supplemental Table 1 and Supplemental Data PILGram_PATTERNS_MDRpred.txt), approximately 12 models from each of the three grammars (PRE, TMR-PRE, and PP-PRE). The models had individual accuracies ranging from 70–75%, underperforming the combination of HMM models that already exist. However, application of the simple voting approach used above in which the number of models that matched each sequence was counted, improved the results dramatically. The accuracy and PPV for increasing numbers of model matches is shown in Supplemental Figure 2, and have maximum values at the most stringent threshold (requiring all patterns be matched) of 99% and 28%, respectively. Using models from each of the grammars individually in the voting approach showed that each grammar, PRE, TMR-PRE, and PP-PRE, performs very similarly in terms of accuracy and PPV when considering the maximum number of model matches (accuracies 96%, 97%, and 95%, respectively, and PPVs all at 12%). From these results it appears that the overall performance of our approach benefits from the combination of different kinds of models, which more than doubles the PPV.

**Figure 2.**
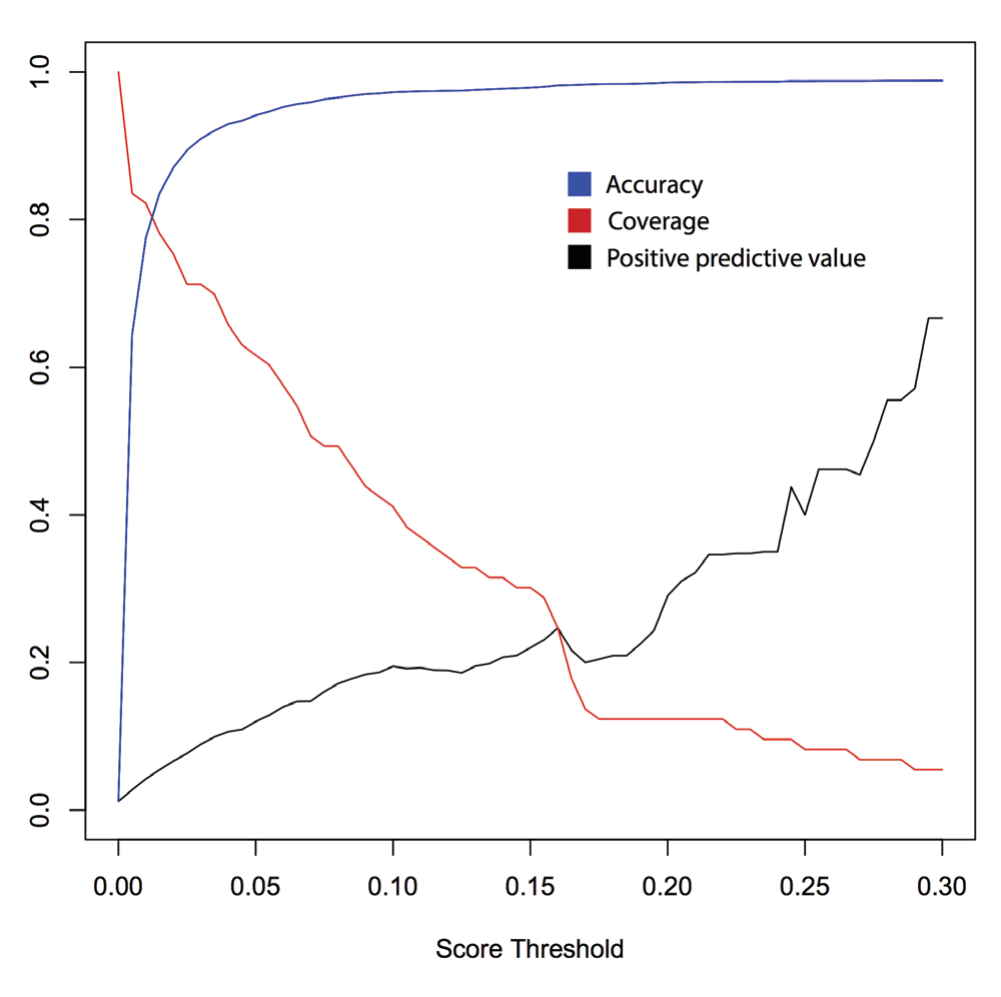
MDR classification results. The accuracy (blue line), positive predictive value (black line), and percentage of total MDR transporters identified (coverage; red line) are shown as a function of the score threshold used (X axis). The score is derived from a logistic regression on the complete set of 36 models generated (see text).

**Supplemental Figure 3.**
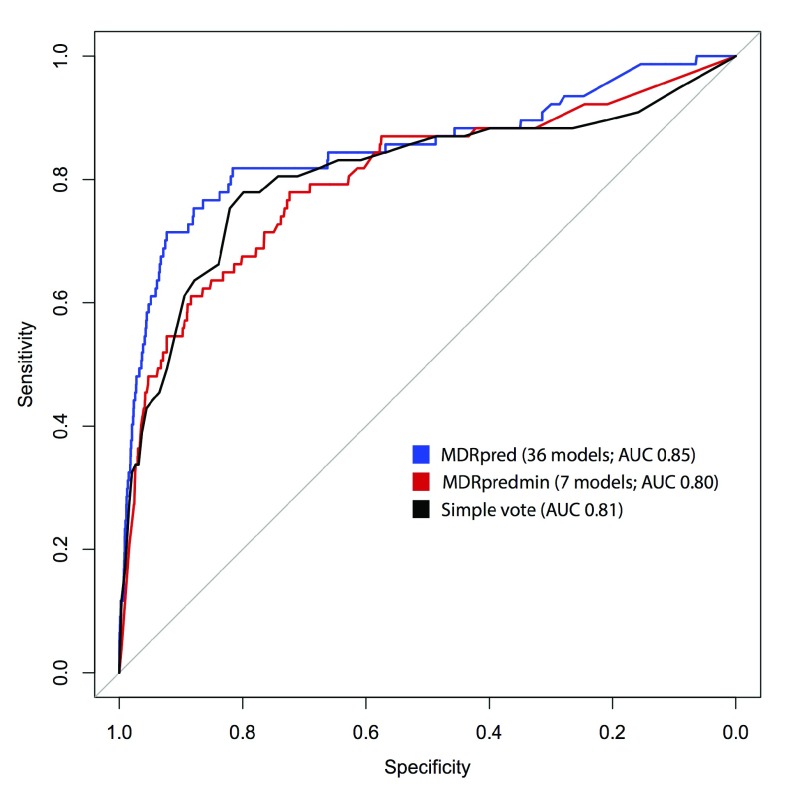
Comparison of MDRpred models. The receiver-operator characteristic curves (ROC) are shown for the simple vote combination of 36 models (black line), the logistic regression combination of 36 models (blue line), and the logistic regression combination of the selected seven models shown in Table 3 (red line). The area under the curve (AUC) for each method is indicated in the legend. The results show that using all models in a score derived by logistic regression provides the best performance, though the other methods also perform adequately.

To examine whether the individual scores could be combined to provide better prediction we employed logistic regression and found that this improved our results somewhat ( Figure 2; Supplemental Figure 3). As a comparison for the same ~97% accuracy level provided by the traditional methods (Pfam family matches) our method, we call MDRpred, identifies 37 of the MDR transporters from our training set (50%) versus 10 for the traditional methods. It is clear that further development is needed to improve classification of this important group, but our approach provides the best method to date of identifying drug resistance transporters using sequence alone.

### Functional motifs identified

In addition to classification of sequences a second goal of this work is to identify biologically relevant regions of proteins that are responsible for protein function. We showed that PILGram can identify regions known to be functionally important in zinc fingers. Here we apply a similar approach to identify regions that may be important for drug resistance in transporters. That is, those regions of the transporter that are most important for their function of transporting a broad class of substrates, antibiotic drugs.

**Supplemental Figure 4.**
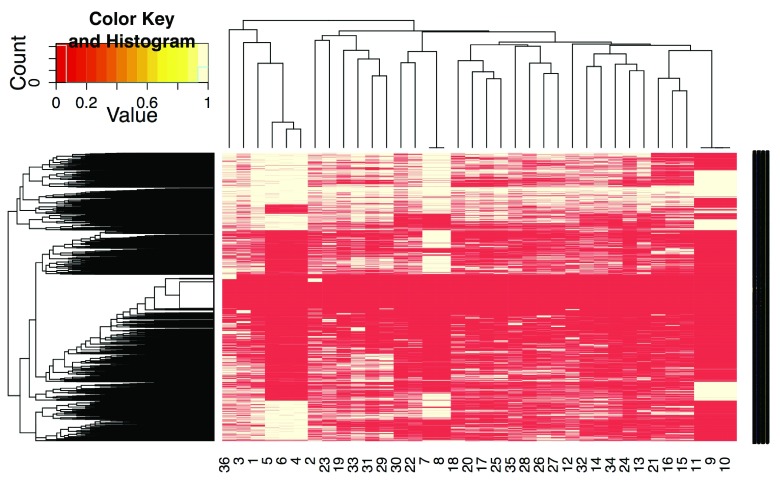
Clustering of MDRpred individual models. For each labeled sequence (rows) we assessed the presence (red cell) or absence (white cell) of a match to any of the 36 MDRpred regular expression models (columns). Hierarchical clustering was used to highlight relationships between models based on their patterns of matches. Dendrograms for sequences and models are shown. The patterns highlight seven groups of models that share a large number of predictions. Representative members from each of these clusters are shown in Table 3.

We first examined the overlap in patterns by clustering models based on the training sequences that they matched ( Supplemental Figure 4). The models were arranged using hierarchical clustering and then seven clusters of similar models were identified. We found that most of the clusters exhibited some similarity in patterns and model from each cluster with the highest independent accuracy listed in Table 5. We found that applying logistic regression to combine these seven models provided a similar performance as the voting method, but underperformed the logistic regression on the complete set of models somewhat ( Supplemental Figure 3). This indicates that the seven models represent a large portion of the information in the approach but that the additional models add significant value.

**Table 5.** Drug resistance transporter patterns identified.

Model	Pattern	PhysiochemicalProp	Accuracy	ClusterName
36	D[^ADGHY]+[AEFHI].+SR		73%	Cluster 1
31	AR.+RL[DMPR-Y]		74%	AR-L
8	AQ.+AT	Solvent Accessibility	73%	AQ-T
18	[AC][DFGLMPQRVY]+RQ		75%	RQ-L
27	[DGLN-V]VR.+TV.+[CDEY]*$		76%	VR
13	AQ.+RQ.{49}		75%	Cluster 6
16	MR.+LL[STVW]		73%	M-L

**Figure 3.**
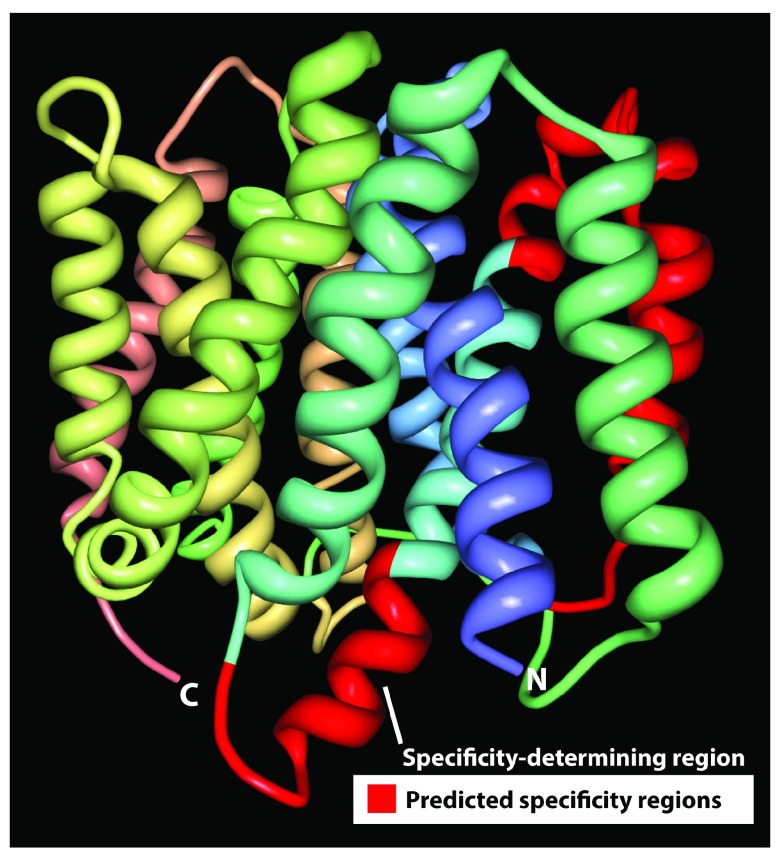
Prediction of selectivity in EmrD. The structure of the MDR transporter EmrD from *E. coli* (2GFP) is shown with the regions of maximum pattern overlap shown in red. This region has been shown to be the substrate selectivity filter for substrates transported by the protein, showing that MDRpred predictions can highlight functionally important regions.

EmrD is an MDR transporter with a solved crystal structure ( Yin *et al.*, 2006). We examined the overlap of the PILGram models on the EmrD sequence and found that the maximum overlap in matched expressions from our models occurred in H3 69-103 and the loop following H4 118-131. The latter region has been highlighted as the ‘selectivity filter’, a loop extending in to the cytoplasm and that abrogates substrate selectivity when mutated ( Yin *et al.*, 2006) ( Figure 3). This suggests that for this case where a substrate selectivity region is known, our model can correctly identify it, though more examples would be necessary to fully demonstrate this. Alignments of matches with individual models with all positive example MDR sequences is provided as Supplemental Data MDRpred_alignments.out.

## Identification of novel MDR transporter candidates from environmental microbiomes

New antibiotic resistance mechanisms are thought to be acquired from a very large natural reservoir of environmental bacteria, most of which have not yet been characterized ( D'Costa *et al.*, 2007; Forsberg *et al.*, 2012; Li *et al.*, 2014). This means that novel antibiotics may face emergence of antibiotic resistance in pathogenic bacteria by lateral gene transfer or other means ( Aminov & Mackie, 2007; Forsberg *et al.*, 2012). We were interested in determining if our models could be used to identify candidate MDRs from environmental samples. We therefore searched a species-resolved metagenomic dataset acquired from consortia ( Cole *et al.*, 2014) cultivated from a phototrophic microbial mat in Hot Lake, Washington ( Lindemann *et al.*, 2013). Though soil microbial communities have been examined for antibiotic resistance potential previously ( D'Costa *et al.*, 2007) communities living in extreme environments such as Hot Lake have not. We postulated that these kinds of communities might be rich sources of novel MDR transporters given the manifold interactions between community members ( Martinez *et al.*, 2009; Piddock, 2006).

We first searched the 69010 protein sequences from the Hot Lake consortial metagenomes (Nelson *et al.*, submitted) for known MDR transporters using the Pfam families (PF00893, PF08370, PF00873, and PF13536) and identified 118 high-confidence (E value < 1e-100) matches. Interestingly, when we examined a set of clones gathered from 18 soil samples and selected for expression of multidrug resistance phenotypes ( Forsberg *et al.*, 2012) we found only 14 MDR transporters at the same stringency, though one caveat is that the efficiency of expression of transporters could be a limitation in this system. This suggests that the Hot Lake community has a relatively large number of MDR transporters.

**Supplemental Figure 5.**
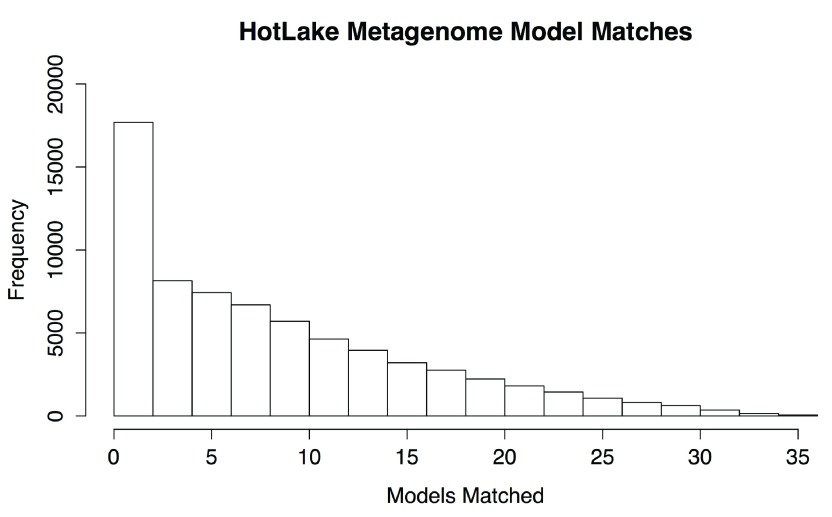
Histogram of MDRpred votes for metagenomic sequences. The number of sequences (Y axis) with N matches (X axis) in the Hot Lake metagenome (69,010 sequences total) is shown as a histogram. This plot can be compared with similar plots in Supplemental Figure 2 showing accuracy and positive predictive value at each of these stringency thresholds.

We believed that there would be MDR in this metagenome that would not be detected using the Pfam families available. Therefore, we searched the Hot Lake consortium metagenome using all 36 models and then ranked the results by number of matched sequences. A histogram of number of matching models is shown in Supplemental Figure 5. Because MDRpred was trained only on transporter proteins it cannot discriminate transporter proteins from non-transporters. That is, there are a significant number of false positive predictions that match proteins unlikely to be transporters. Accordingly, we filtered candidates to only those proteins identified as transporters by Pfam (list of Pfam transporter families provided as Supplemental Data Pfam_transporters.txt) and at the highest stringency we identified five candidate MDR sequences ( Table 6). This step is included in the overall MDRpred process to allow accurate prediction in entire genomes or metagenomes. We provide a full list of other high-confidence predictions (matching more than 30 individual models, annotated as transporters but not multidrug resistance transporters by Pfam) as Supplemental Data HotLake_MDRpred_predictions.fasta.

Though two of these predictions are already annotated as transporters (arabinose efflux permease and lipid transporter) these are largely automated predictions based on traditional sequence analysis approaches (BLAST searches and family/motif matches). Novel antibiotic resistance transporters are likely to show some similarities with known transporters ( Forsberg *et al.*, 2012), but definite substrate specificity is often not revealed by these relationships. The value of MDRpred is the potential to identify novel antibiotic resistance transporters from sequences annotated as transporters where substrate specificity has not been experimentally established.

**Table 6.** Predicted novel multidrug resistance transporters from Hot Lake.

ID	Description	Length
CY41DRAFT_3272	Arabinose efflux permease family protein	434
HLSNC01_00824	ATPase components of ABC transporters	547
HLSNC12_00368	Putative oligoketide cyclase/lipid transport protein	152

## Discussion and conclusions

The explosion in number of sequences available from a large number of sources has driven the need for better methods to capture patterns in distinct groups of functionally related sequences. Our method, based on linguistic approaches to pattern identification, has several advantages over existing methods. Not requiring a sequence alignment means that important and discriminatory sequence regions can be identified from functionally similar proteins that may be highly evolutionarily divergent or where the evolutionary relationships are unclear. Having a wide range of grammars that can be applied in the framework is a significant strength, allowing for flexible pattern discovery. In the current paper we use only variants of a protein regular expression grammar, but other grammars can easily be used depending on the application. For example, context-free grammars could be applied to better identify potential non-local interactions between different regions in the protein sequences.

In the current study we have shown that PILGram can be successfully applied to identify patterns in proteins sequences, first by application to known functional sequences from the PROSITE database, and then by application to a set of proteins related by function but where functional determinants of specificity are not well understood. From our initial work with PROSITE families we found that some kinds of patterns may be more amenable to identification using PILGram, but this was a limited proof-of-concept application that would merit further characterization. In the case of the zinc finger pattern, which has variable spacing between active cysteine and histidine measurements we found that very accurate models could be obtained by taking a simple voting approach between multiple independent PILGram models.

Application of our approach to the MDR sequences identified a set of over 30 individual PILGram models that, when combined, provided very good accuracy and positive predictive value, relative to a combination of existing HMM models in Pfam. To our knowledge this is the first attempt to develop a predictive model for MDR transporters across families. Similar to our results with PROSITE patterns we found that these models could identify regions known to be important for substrate specificity in MDRs. This represents a step forward in classification of this important group of transporters.

The vast number of uncharacterized and often unculturable bacteria in environmental communities represent a large amount of genetic potential given the ability of bacteria to share genetic information. As an example application, we ran our method on sequences identified from a moderately complex community derived from an extreme environment, in this case the Hot Lake unicyanobacterial consortia ( Cole *et al.*, 2014). We identified five candidates that were strongly predicted by the combination of our models to be MDRs. Given that the positive predictive value of the combined method is nearly 30% it is likely that one or two of these predictions is a true positive. Further research is needed to be able to predict specific drug substrate specificities for MDRs and other transporters.

We believe that the method we describe, MDRpred, will complement well the other commonly used sequence annotation methods and that it provides a unique set of predictions about potential novel MDRs. Furthermore, the PILGram approach to identification of functional patterns in unaligned sequences has applications in a large number of other problematic protein groups where function is conserved over sequence.

## Data and Software availability

### Software access

A publication describing the PILGram software is currently in preparation (Gosink & Bruillard, manuscript in preparation) but the software is available upon request from the authors.

### Latest source code

Code implementing the MDRpred algorithm as described is available on Github ( http://github.com/biodataganache/MDRpred).

### Source code as at the time of publication

https://github.com/F1000Research/MDRpred/releases/tag/V2.0

### Archived source code as at the time of publication

http://dx.doi.org/10.5281/zenodo.17514

### Software license

Apache License v2.0

***Figshare:*** Prediction of multi-drug resistance transporters dataset doi: 10.6084/m9.figshare.1415804 ( McDermott *et al.*, 2015).

## Figures and Tables

**Supplemental Table 1.** PILGram models for MDR transporter prediction.

Order	PP	RESmall	Accuracy	Cluster	ClusterName	Grammar	RE
1	_SecondaryStrD1075	.{408}$	70%	1	Cluster 1	PP-PRE	.{408}$
3	_SolventAccessibilityD3075	LM.*VV	72%	1	Cluster 1	PP-PRE	LM.*VV
4	_ChargeD1100	VG.*GL[^CQ].{233}	72%	1	Cluster 1	PP-PRE	VG.*GL[^QC].{233}
5	_PolarityD3100	VG.*GL[^EF].{233}	72%	1	Cluster 1	PP-PRE	VG.*GL[^FE].{233}
6	_HydrophobicityD3100	VG.*GL[^DH].{233}	71%	1	Cluster 1	PP-PRE	VG.*GL[^HD].{233}
**36**	**NA**	**D[^ADGHY]+[AEFHI].+SR**	**73%**	**1**	Cluster 1	PP-PRE	**D[IK-SCFK-WE]+[^YC-DQWK-WG].+SR**
2	_SecondaryStrT13	GR[^A-P]	70%	2	AR-RL	PP-PRE	GR[^PA-PA]
19	NA	AR.+AP[DGILNPQRVW]	74%	2	AR-RL	TMR-PRE	AR.+AP[^YAKASHKTMEHCKTEYMSECMYF]
23	NA	RT.*AG.+$	66%	2	AR-RL	TMR-PRE	RT.*AG.+$
29	NA	[RSTVWY]*.{41}AR.+RL	71%	2	AR-RL	PRE	[^EA-IEGH-NAAQ]*.{41}AR.+RL
**31**	**NA**	**AR.+RL[DMPRSTVWY]**	**74%**	**2**	AR-RL	PRE	**AR.+RL[PM-MDUR-YM]**
33	NA	AR.+LR	70%	2	AR-RL	PRE	AR.+LR
7	_NormalizedVDWVD2001	AQ.+AT	73%	3	AQ-T	PP-PRE	AQ.+AT
**8**	**_SolventAccessibilityD1100**	**AQ.+AT**	**73%**	**3**	AQ-T	PP-PRE	**AQ.+AT**
22	NA	AQ.*LF.*LT.*	72%	3	AQ-T	TMR-PRE	AQ.*LF.*LT.*
30	NA	AQ.*TI[AQRSTVWY]+	71%	3	AQ-T	PRE	AQ.*TI[^LC-P]+
17	NA	RQ.*LA[ACDE]	73%	4	RQ-L	TMR-PRE	RQ.*LA[^TF-YM]
**18**	**NA**	**[AC][DFGLMPQRVY]+RQ**	**75%**	**4**	RQ-L	TMR-PRE	**[^DM-YYRL-VEUQ][GG-GFVMQD-DLUQ-** **RMPY]+RQ**
20	NA	RQ.+LL[AEFHKNRT]	74%	4	RQ-L	TMR-PRE	RQ.+LL[^PGDV-WSDM-QPYI-LC]
25	NA	RQ.*LA.{12}[^GM-Y]*[^MY]{14}	71%	4	RQ-L	PRE	RQ.*LA.{12}[^M-YG]*[^YM]{14}
26	NA	[KLNPQSTVW]VR.*AV	72%	5	VR	PRE	[^RA-IIEKCMAFIM]VR.*AV
**27**	**NA**	[DGLNPQRSTV]VR.+TV.+[CDEY]*$	**76%**	**5**	VR	PRE	[LGDN-VU]VR.+TV.+[^QF-NUAF-WH]*$
28	NA	[^D-LW]+VR.+ML	71%	5	VR	PRE	[^MD-LW]+VR.+ML
35	NA	[^A-KNR]SR.*AA[ADEGHIKSTVY]+	72%	5	VR	PRE	[^NA-KR]SR.*AA[^FF-FLWL-QCFI-RK]+
12	NA	RV.+IA.[K-S]	72%	6	Cluster 6	TMR-PRE	RV.+IA.[MK-SS]
**13**	**NA**	**AQ.+RQ.{49}**	**75%**	**6**	Cluster 6	TMR-PRE	**AQ.+RQ.{49}**
14	NA	NV.+AQ.{48}	70%	6	Cluster 6	TMR-PRE	NV.+AQ.{48}
24	NA	VV.+VR.*RQ.*LA*[ACDT]	72%	6	Cluster 6	PRE	VV.+VR.*RQ.*LA*[^WE-SC]
32	NA	ID.+AQ.{48}	70%	6	Cluster 6	PRE	ID.+AQ.{48}
34	NA	.{45}AQ.+LV.*AR.*FN*	74%	6	Cluster 6	PRE	.{45}AQ.+LV.*AR.*FN*
9	_SolventAccessibilityT12	MF.*QL	72%	7	M-L	PP-PRE	MF.*QL
10	_NormalizedVDWVT13	MF.*QL	71%	7	M-L	PP-PRE	MF.*QL
11	_PolarizabilityT12	MF.*QL	71%	7	M-L	PP-PRE	MF.*QL
15	NA	MR.+LV.{49}.{49}	71%	7	M-L	TMR-PRE	MR.+LV.{49}.{49}
**16**	**NA**	**MR.+LL[STVW]**	**73%**	**7**	M-L	TMR-PRE	**MR.+LL[^HA-RD]**
21	NA	MR.+AQ	71%	7	M-L	TMR-PRE	MR.+AQ

**Table:** 

Columns are as follows	
Order	The order the pattern was generated (no significance)
PP	If the pattern includes physiochemical properties this column indicates which property was used. NA indicates no PP was used.
RESmall	A canonical regular expression generated by PILGram (non- canonical expressions are in column labeled RE)
Accuracy	The cross-validated accuracy of this pattern
Cluster	The cluster assignment from clustering patterns based on which examples were matched
ClusterName	A descriptive name for the cluster
Grammar	The original grammar used for generation of the pattern. Note that TMR-PRE will result in similar patterns as PRE because it simply biases the search space at the outset
RE	The original regular expression generated by PILGram. This is functionally equivalent to the RESmall, but frequently contains redundant character sets.

**Table:** 

PPs included	
_ChargeD1100	Distribution of positively charged amino acids [KR]
_HydrophobicityD3100	Distribution of hydrophobic amino acids [CLVIMFW]
_NormalizedVDWVD2001	Distribution of medium-sized amino acids [NVEQIL]
_NormalizedVDWVT13	Transitions between small and large amino acids [GASTPD] <->[MHKFRYW]
_PolarityD3100	Distribution of highly polar amino acids [KMHFRYW]
_PolarizabilityT12	Transition between low and medium polarizable amino acids [GASDT]<->[CPNVEQIL]
_SecondaryStrD1075	Distribution of amino acids that tend to form alpha helices [EALMQKRH]
_SecondaryStrT13	Transition between helical and coil amino acids [EALMQKRH]<->[GNPSD]
_SolventAccessibilityD1100	Distribution of amino acids that tend to be buried [ALFCGIVW]
_SolventAccessibilityD3075	Distribution of amino acids that tend to be intermediate between buried and exposed [MPSTHY]
_SolventAccessibilityT12	Transition between buried and exposed amino acids [ALFCGIVW]<->[RKQEND]
