# Supplementary material for: Prediction of multi-drug resistance transporters using a novel sequence analysis method
Source: F1000Res. 2015 May 29;4:60. Originally published 2015 Mar 9. [Version 2] doi: 10.12688/f1000research.6200.2 (PMC4743146; doi:10.12688/f1000research.6200.2)
Supplement: Peer review history

# Peer review: Referee response for version 2

**DOI:** 10.5256/f1000research.6999.r8819

**Published:** 2015-06-17

**Authors:** David Baltrus

**Affiliations:** 1 School of Plant Sciences, University of Arizona, Tuscon, AZ, USA

It's good to go, my critiques have been adequately addressed .

I have read this submission. I believe that I have an appropriate level of expertise to confirm that it is of an acceptable scientific standard.

# Peer review: Referee response for version 2

**DOI:** 10.5256/f1000research.6999.r8818

**Published:** 2015-06-01

**Authors:** Robert Flight

**Affiliations:** 1 Resource Center for Stable Isotope-Resolved Metabolomics, University of Kentucky, Lexington, KY, USA

Much improved, and much clearer how the PILGram regex's are being generated and used.

I have read this submission. I believe that I have an appropriate level of expertise to confirm that it is of an acceptable scientific standard.

# Peer review: Referee response for version 1

**DOI:** 10.5256/f1000research.6648.r7890

**Published:** 2015-03-25

**Authors:** David Baltrus

**Affiliations:** 1 School of Plant Sciences, University of Arizona, Tuscon, AZ, USA

Given the growing problem of antibiotic resistance across bacterial pathogens, Multi Drug Resistant (MDR) transporters are an intrinsically important group of bacterial proteins. However, unlike other resistance protein families where precise characterization is possible (i.e. B-lactamases), and while we can often "see" MDR transporters in bacterial genomes due to sequence similarity, it is nearly impossible at the present time to accurately annotate what antibiotic substrates these transporters act on. As genome sequences pile up, and whole genome sequences begin to be used to predict drug resistances/sensitivities in clinical settings, the it becomes increasingly important to accurately describe the roles of MDR transporter complexes.

The article from McDermott *et al*. focuses on using grammar based alignment free approaches in order to predict and classify MDR protein complexes, and develops a program called PILGram for this purpose. The authors do a good job of describing the problem they are addressing throughout the introduction, and giving examples of the utility of grammar based approaches to an audience that is likely not well versed in these analyses. Realistically, the results are not exceptional. The model does a great job of predicting ser/thr phosphatase patterns, but current approaches using similarity based searches do a pretty good job as well. The model does slightly worse than conventional methods with the prediction of zinc-fingers, likely because of their unstructured nature, but taking the consensus using grammar based approaches is still on par with other widely used methods. The authors don't improve on predictions for these two classes with grammar based methods, but they provide a good demonstration that such models can work on par with conventional analyses. I think it's important to develop both sequence based and sequence independent approaches and that these go hand in hand rather than act in a mutually exclusive way.

The rubber meets the road when the authors try to predict novel MDR classes, and the results are not great. While numerically, the data seems to show that PILGram is able to be trained to identify MDR transporters with levels of accuracy above randomness, it misses a lot. On the other hand, so do conventional analyses, which is what makes this an interesting problem to tackle. Moreover, the authors use a metagenomic dataset (nicely done by the way, I wasn't expecting that) to try and predict novel MDR transporters. The data do suggest that PILGram can pick up *something* of a signal within these metagenomes compared to a soil sample, which is encouraging. However, I'm left with a bit of an unenthusiastic taste in my mouth when I see the table of "high confidence novel transporter proteins" and 3 of the 5 are annotated as some kind of transporters, and the other two are FtsH and a related protein. The authors do point out that it's likely that at least one of these is a true positive (my guess, it's not either of the last two), but it would be good in the discussion if the authors could further flesh out what differentiates the data that PILGram is giving you from simply looking through the annotations for "transporter" proteins given that 3 of 5 are likely transporting something based on the JGI annotation. Said more plainly, it would be good if the authors could describe what PILGram is telling them about the first three genes in table 4 that the annotations don't. I think this would really wrap the story up better.

My overall impression is that this is a solid paper, albeit without really exceptional results. However, utilization of these sequence alignment independent grammar models and pipelines and descriptions for how they behave on real world data is a step forward and therefore worthy of being published. The data is solid, and the authors do a good job of describing the limitations. We need anything and everything possible to be able to predict MDR proteins given the large amounts of genome data that are going to be piling up. PILGram will only get better with larger training sets.

Slight side note...I'm wondering whether glc-1 from *C.elegans* should be included in the training set for the "Prediction of multi-drug resistance transporters dataset" table. Seems weird to me given that these are bacterial proteins.

I have read this submission. I believe that I have an appropriate level of expertise to confirm that it is of an acceptable scientific standard, however I have significant reservations, as outlined above.

# Response

**Published:** 2015-05-08

**Authors:** Jason McDermott

**Affiliations:** Department of Computational Biology, Pacific Northwest National Laboratory, USA

We thank both the reviewers for their insightful and very helpful comments. We have revised the manuscript according to the reviewers’ suggestions and feel that it is substantially improved in terms of clarity and potential for reproducibility. Importantly, we have provided more complete data, results, and code that we employ in the paper.

Dr. Baltrus points out that two of the high-confidence predictions made by the method are annotated as non-transporter proteins. This issue arises from the fact that a preliminary screen was done on the metagenome to identify transporters (in the ‘Identification of novel MDR transporter candidates from environmental microbiomes’) using Pfam families. As explained in the paper this is necessary because MDRpred was trained only on transporter proteins and so may give spurious results when applied to non-transporters. However, it looks like the FtsH sequence was erroneously included as a transporter- probably because it has an ABC-associated ATPase domain. The other protein, listed as a “Bacterial cell division membrane protein”, has a membrane domain associated with O-antigen, but this appears to be involved in synthesis of O-antigen and not transport. These Pfam families have both been removed from our list for transporters, which is now provided as a data file. Table 4 (now Table 6) has been updated by removing these two predictions and a complete set of higher-confidence predictions is now included as Supplemental Data file.

Dr. Baltrus also asked for clarification of what MDRpred would be giving beyond examining annotations for “transporter” proteins. The value of MDRpred is that it will predict which transporter proteins are capable of transporting a specific class of substrates, antibiotic drug compounds. As we point out in the text this is a broad class of compounds and is often incompletely defined for individual well-studied transporters. However, out method is able to accurately classify MDR transporters *relative* to other transporters that do not transport drug compounds. Even in the case of the first and third predictions (annotated as arabinose and lipid transporters, respectively) the specific annotations are based on best matches by Pfam or BLAST, and may not accurately represent the substrates that are actually transported depending on how close the matches are. We now include an extended discussion of the interpretation of the list of proteins found in Table 4 (now Table 6) in the Results section.

Glc-1, a glutamate gated transporter, can confer drug resistance in *C. elegans*, though it appears that it does not transport drugs itself. We’ve removed it from the training set.

# Peer review: Referee response for version 1

**DOI:** 10.5256/f1000research.6648.r7889

**Published:** 2015-03-19

**Authors:** Robert Flight

**Affiliations:** 1 Resource Center for Stable Isotope-Resolved Metabolomics, University of Kentucky, Lexington, KY, USA

**Claims**

- Implement a linguistic-based approach that allows the identification of functional patterns from groups of functionally related proteins that does not require alignment of the proteins
- The method uses regular-expressions that are generated using a parse-tree that is modified via a genetic algorithm, and fitness is scored by accuracy using training data.
- Able to find discriminative patterns for serine-threonine phosphatases, zinc fingers, and multi-drug resistance (MDR) transporters
- Predict MDR transporters in a bacterial community from "Hot Lake" as a potential pool for novel MDR transporters that could be transferred to current bacteria as novel source of antibiotic resistance
- PILGram is able to identify and separate based on the binding region responsible for substrate specificity

**Praises**

From this version of the manuscript (v1), the claims are justified. Regular expressions are a linguistic construct, the authors are able to reproduce previously defined regexes without prior alignment using the PILGram method, and classify zinc fingers and MDRs by counting the number of regexes matching a particular sequence, resulting in the MDRPred method. This method, MDRPred was then applied to a newly sequenced bacterial community and possibly novel MDR transporters identified.

In addition, from the text, the generation and validation of the regexes was done in a statistically rigorous way, with half of the data used for training and half of the data used for testing / validation / calculation of metrics. This is nice to see in this kind of paper, as it has become the exception rather than the rule.

**Reservations**

Although I think the general claims can be justified from the text, there are some areas of concern that I think should be addressed in a subsequent version of the manuscript. 

These reservations fall under these major areas, ordered in what I consider most important to least important:

- data availability for reproducibility
- actual MDRPred code
- actual id's for positive and negative examples
- Weak "substrate specificity" claim
- lack of description in the text leading to either lack of clarity or possible misunderstandings
- PILGram details
- Physiochemical Properties and TMR
- Elaboration of clustering
- Supplemental Table 1
- Describing REGEXE's
- language implying other methods are not "linguistic"

Each of these reservations are further detailed below.

**Data & Code Availability**

Not all of the code / data necessary to reproduce the results are currently provided. While acknowledging that the primary algorithm (PILGram) is currently awaiting publication and that this is **not** the place to describe the particulars of that software, I think there are still steps to be taken to improve the reproducibililty of **this** publication by providing more of the data. It should be noted that when the PILGram algorithm is published, this publication should be updated with references and links to make it easier for others to find. 

That being said, this is a publication about a **method**, and although the particulars of the **method** are well described, there is no accompanying code, scripts, even psuedocode supplied so that the reader might make use of the **method** themselves, either on the provided supplementary FASTA files, or on their own sequences. I searched github for the term "mdrpred", and also for the lead authors' name and twitter username to no avail. The need for an actual script or executable (preferably open source) is increased after reading the description of including PP-PRE and TMR-PRE, and calculating their matches, as this section is a little unclear as to how exactly that calculation is performed with no example (see comment below).

In addition to the code, other data that should be included are:

- UniProt entries for positive and negative examples for serine-threonine phosphatases and zinc fingers
- genome accession and gene annotations from the metagenome analysed
- list of metagenome accessions annotated as MDR's using MDRPred
- Date of download of PROSITE data. prosite.dat on Mar 17, 2015 shows 198 positive matches for PS00125, and I'm assuming 2018 (hard to tell from file) positive matches for PS00028, versus 166 and 1997 sequences mentioned in the text.
- Text files of the regexes generated by PILGram in each case

**Weak "substrate specificity" claim**

This is mentioned in the abstract, and 2 times in the introduction. The wording in the abstract implies that the method is able to delineate substrate specificity, i.e. that the method can generate regexes that are specific for different substrates. However, the one result implies rather that the regexes identify the region responsible for substrate specificity (which is really neat). These seem to be two different things in my mind, and I think either the claim in the abstract and introduction should be dropped or clarified, especially given that there is only one example provided. Finally, the claim is further weakened in the current text because the word **substrate** is missing from the paragraph discussing the evidence for substrate specificity (Results, Drug resistance transporters, Functional motifs identified, last paragraph in that section, no mention of "substrate", just specificity).

More so than the "substrate specificty" claim, I think the authors would do well to place more emphasis on the fact that *all* of this work is done on sets of sequences **without alignment** first! It might just be me, but this was to me one of the most important things in the paper (and something I will probably make use of in my own research), that did not seem to be highlighted enough.

**Lack of Description**

**PILGram Algorithmic Details**

Again I acknowledge that this is not the place to detail the full inner-workings of the PILGram algorithm, and the example in the text for BMI is useful. However, most genetic algorithms have a defined chromosome length defining the solution. I would have expected an analogous situation for PILGram, in that one would have to define the **length** of the regular expression. This does not appear to be the case here, given the variety of reg-ex's noted for Zinc fingers and MDRs. As far as I can tell, this is likely due to the way that individual trees can be recombined, but it is not clear from the text how different length regexes result. Clarification of how different length regexes result would be useful.

**Physiochemical Properties and TMR**

I think I understand why the physiochemical properties (PP) and transmembrane region (TMR) score were included for the MDRPred, however there is currently no discussion of their inclusion or justification in the text. From the current description of them, it is also difficult to imagine how something matches the PP-PRE and TMR-PRE, including the PP and TMR scores as part of the match. Therefore I recommend:

- Having a better description of the PP and TMR scores in Methods
- Justification for the inclusion of PP-PRE and TMR-PRE in MDRPred. Currently the only justification is "Because we believed ...". I would hazard a guess that the accuracy drops precipitously without them, but there is nothing in the text currently describing why they are needed.
- Giving examples of how some PPs are different for different AAs
- Example of calculation of PP score and TMR score for a regex match
- Example of full match for a derived PP-PRE or TMR-PRE

**Elaboration of clustering**

In the Results, "Functional motifs identified", a description of clustering the generated models is provided. The current description is ambiguous. I think what was done was a vector of length 71 (corresponding to the number of training sequences) was generated for each model, with a 1 indicating a match to the model, and 0 indicating no match to the model. These 36 vectors (one for each model) were subsequently clustered using hierarchical clustering. 

No description of what distance metric was used to calculate the distance between the model vectors, nor which hierachical clustering method was used is provided. In the R stats package, there are: two variations of Ward's minimum variance method, the complete linkage method, the single linkage method, median, and centroid. The software, version, and algorithm reference should be provided for completeness.

Supplemental Figure 4 should have the clusters indicated on the figure (boxes or something).

**Supplemental Table 1**

I believe supplemental table 1 could benefit from including:

- a description of what each column is beyond the title (for example, what is the difference between RESmall and RE??)
- a description of the PP that are included (it appears there are only 11 that end up being used)
- an indication of which are PRE, PP-PRE, and TMR-PRE

**Describing REGEXE's**

I use regular expressions regularly, but even still I found it difficult to follow the regular expressions listed in the text without looking to a reference. A short description, even in the supplemental materials of general features of the regexes would be useful. For example, the fact that [ABC] means one of either A or B or C at that position, that {3, 8} means either 3 or 8 letters between the previous and the next thing, and that [^ABC] means none of either A or B or C.

Further, having examples of what portion of a sequence is matched, especially for the serine-threonine case where the sequence interval in general overlaps between the PROSITE pattern and the PILGram derived pattern. But also having examples for the Zinc finger showing the attributes shown, or describing what part of the regex encodes which features would help a lot.

**Language implying other methods are not linguistic**

In its current form, the abstract reads:

"In this paper we describe a linguistic approach to identify ..."

This implies that regular expressions in PROSITE and hidden markov models are not "linguistic" approaches. However, in the text, describing regular expressions used by PROSITE as the simplest form of grammar (regular grammars), and Hidden Markov models as a type of regular grammar (Introduction, paragraph 3). If these are grammars, then that implies they are linguistic approaches. 

In fact, from the description in the manuscript, PILGram generates regular expressions that in some cases are very similar to those used by PROSITE. It seems currently unclear as to how generating regular expressions using PILGram is a "linguistic" approach, but aligning and finding common features (as in PROSITE or HMMs) is not.  

I understand that PILGram is able to generate discriminative regexes without alignment first, and that is very useful (as exemplified by this manuscript), but from the current description that does not make it "linguistic". I admit I may be missing something in reading the current text in this area, as I am not a linguist.

**Other Simple Improvements**

Methods: under PILGram, first sentence, a reference is missing to SIEVE.

Methods: PILGram example, example grammar, spaces around symbols would greatly 

improve the readability

In Results: actually identify the **core** of the PROSITE reg-ex that PILGram

is able to capture, noting that PILGram drops the first and last AA in the PROSITE

one, and adds **Q** to the set of alternatives compared to PROSITE.

Results: paragraph 2 says "Supplemental Table 1", but I believe this should be

"Supplemental Figure 1".

Results: "We found that applying logistic regression to combine these seven models provided a similar performance as the voting method, but underperformed the logistic regression on the complete set of models." **how much** did it underperform, curious minds want to know??

Methods: hot lake peptide data, the wording implies single bacterium genome, however, the **Results** makes it clear that a metagenome is being used. One of these two sections should be modified to clarify whether it was a single genome or a metagenome. In addition, if these sequences have been submitted, accession numbers should be provided.

I have read this submission. I believe that I have an appropriate level of expertise to confirm that it is of an acceptable scientific standard, however I have significant reservations, as outlined above.

# Response

**Published:** 2015-05-08

**Authors:** Jason McDermott

**Affiliations:** Department of Computational Biology, Pacific Northwest National Laboratory, USA

We thank both the reviewers for their insightful and very helpful comments. We have revised the manuscript according to the reviewers’ suggestions and feel that it is substantially improved in terms of clarity and potential for reproducibility. Importantly, we have provided more complete data, results, and code that we employ in the paper.

Dr. Flight had a number of points grouped by subject matter so I’ve addressed each of them below using the same organization.

**Data and code availability**

Dr. Flight’s points are very good. We now include the requested datasets in the manuscript and reference them appropriately (see below for details). We have put a script and associated files on Github that represents the MDRpred algorithm as an open source project at: https://github.com/biodataganache/MDRpred

The following data files have been added to the manuscript:

1. UniProt ids and sequences have been included for both positive and negative examples for the PS00125 and PS00028 PROSITE patterns.
2. Genome accessions and links to genome annotations for all sequenced genomes in the metagenome used have been provided. Sequence bins (that is, sequences that are specific to a species that hasn’t been sequenced as an axenic culture) are currently being deposited in GenBank and the manuscript will be updated when accession numbers are available.
3. A full list of high-confidence MDR predictions from the metagenome and their annotations are provided.
4. The original PROSITE data records used in our analysis are provided. These both have been updated in PROSITE since our analysis and the numbers of sequences changed then.
5. The lists regular expressions associated with each problem (the two PROSITE patterns and the MDR task) are now provided as text files.
6. As soon as the PILGram code is released we will update the manuscript with a link to the software and citation for the publication.

 

**Weak substrate specificity claim**

We have updated the manuscript in several places (Introduction, Results, and Discussion) to clarify our claim of substrate specificity. MDRpred predicts substrate specificity at a broader class level, essentially drug-type compound or not. We have included text to explain this distinction and also updated our discussion of specificity in the Results section to make clear that we mean substrate specificity at this broad level.

We have also added stronger language about the lack of need for sequence alignment for our method to work, which we agree is one of the major points in the paper.

**PILGram Algorithmic details**

In the Methods section we include a paragraph describing how the genetic algorithm operates on parse trees. It is indeed the case that the length of the regular expression is not fixed because genetic algorithm recombinations occur on these trees. We believe that this, combined with the other clarifications of the method now included in the revision, should adequately resolve this confusion.

**Physiochemical Properties and TMR**

To address Dr. Flight’s comments we have greatly expanded our description of how the physicochemical properties and TMR scoring and grammars work. Also we have examined the contribution of models arising from each of these grammars to the overall method performance. Interestingly, we found that models from each grammar displayed very similar performance independently and each contributed to the final combined performance. We now provide examples of matches and of how the scores are calculated for different sequences and for different physicochemical properties.

This was a good suggestion and we believe that the manuscript is really strengthened with these revisions.

**Elaboration of clustering**

The details of the clustering approach are now described in a new Methods subsection, “ **Pattern clustering”**.

**Supplemental Table 1**

Supplemental Table 1 now includes column descriptions, descriptions for the PPs that were included, and a column indicating the source of each pattern (PRE, TMR-PRE, or PP-PRE).

**Describing REGEXE’s**

We have added a subsection to the Methods titled, “Regular Expressions”, which summarizes interpretation of the regular expressions found in the manuscript.

We have also added examples for the PROSITE patterns showing which portions of sequences were matched by the PILGram-generated patterns as new Tables 2 and 4 the Results section. The full alignment files are now provided as supplemental data files.

**Language implying other methods are not linguistic**

Dr. Flight’s point is well-taken. It was not our intent to imply that other approaches, like those we mention (HMMs and PROSITE), are not derived from linguistics. We have revised the text throughout to make clear that other currently used bioinformatics methods are also derived from linguistics.

**Simple improvements**

All addressed.
